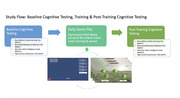# Psychosocial Well‐Being Factors and Adherence as Predictors of a Working Memory Game’s Outcomes in Older Adults

**DOI:** 10.1002/alz.089991

**Published:** 2025-01-09

**Authors:** Micaela Noelle Andreo, Francisco J Sierra, Paulina Skolasinska, Evan T. Smith, Chandramallika Basak

**Affiliations:** ^1^ University of Texas at Dallas, Richardson, TX USA

## Abstract

**Background:**

Preventative interventions for cognitive decline are crucial as the number of individuals with Dementia is projected to reach 78 million by 2030. Cognitive Training can be a promising solution for the maintenance and improvement of neurocognitive functioning and has the potential to delay the onset of AD. This study utilizes a home‐based gamified cognitive training, the Bird Watch Game Unity (BWGU), where participants engage aspects of cognitive control to focus on predictable and unpredictable probe‐cue sequences of novel visual stimuli – pictures of birds. This game trains skills such as updating, focus switching, and working memory capacity.

**Method:**

Fifty‐six healthy older adults (>65 years) played the BWGU for approximately 15.25 hours on average. Adherence is defined as frequency (the average lagged days between sessions) and duration (total hours trained). This study’s purpose is to determine if fluctuations in daily well‐being measures such as sleep, mood, stress, wellness, and busy‐ness influence a participant’s adherence. Predictive relationships between an individual’s psychosocial well‐being to adherence and their game learning outcomes will also be discussed. Autoregressive Integrated Moving Average (ARIMA) models are used to forecast a participant’s training adherence from day‐to‐day, using their previous days’ relationship with well‐being measures. Secondly, the well‐being and adherence measures are used to predict changes in cognition between pre‐ and post‐training visits at the group level via multiple regression. The cognitive battery being compared at pre‐ and post‐ include RAVLT, RBMT, WAIS‐V, and DSST. Lastly, we will explore the relationship between adherence to the BWGU and well‐being measures to determine if the BWGU has a significant impact on older adult’s well‐being, in addition to the cognitive benefits.

**Result:**

For approximately 30% of participants, the ARIMA models of well‐being and adherence predicted daily average scores. For some individuals, well‐being measures were sufficient alone, and for others the addition of adherence improved their model’s prediction of game outcomes.

**Conclusion:**

Overall, these findings provide valuable insight into psychosocial factors of learning and adherence to cognitive training, which in turn would help determine both who would benefit the most from training, and the necessary changes to improve future cognitive training interventions.